# Ménétrier-like disease in a Pointer with concurrent granulomatous gastritis, helicobacteriosis and leishmaniosis: a case report

**DOI:** 10.1186/s12917-021-02802-3

**Published:** 2021-03-02

**Authors:** E. Lagerstedt, T. Spillmann, N. Airas, L. Solano-Gallego, S. Kilpinen

**Affiliations:** 1 Department of Equine and Small Animal Medicine, Faculty of Veterinary Medicine, University of Helsinki, P.O. Box 57, FI-00014 Helsinki, Finland; 2 Department of Veterinary Biosciences, Faculty of Veterinary Medicine, University of Helsinki, P.O. Box 57, FI-00014 Helsinki, Finland; 3 Departament de Medicina i Cirurgia Animals, Facultat de Veterinària, Universitat Autònoma de Barcelona, Bellaterra, 08193 Barcelona, Spain

**Keywords:** Ménétrier’s-like disease, Menetrier's disease, Giant hypertrophic gastritis, *Helicobacter* spp., *Leishmania*, Foveolar hyperplasia

## Abstract

**Background:**

Ménétrier-like disease is a rare hypertrophic canine gastropathy, reported in only seven dogs. Clinical signs are vomiting, anorexia and weight loss. Macroscopically, giant cerebriform gastric mucosal folds are typically seen in the corpus and fundus of the stomach. Histopathologically, fundic mucous cell hyperplasia and loss of parietal and chief cells are typical.

**Case presentation:**

A nine-year-old spayed female Pointer had a history of intermittent vomiting, marked weight loss and hypoalbuminaemia. A gastroduodenoscopy was performed three times within three months with macroscopic changes remaining the same. The gastric mucosa of the corpus, fundus and proximal antrum was markedly irregular, with cerebriform mucosal folds. In the first gastric biopsies, histopathology revealed a moderate granulomatous gastritis, with a severe manifestation of *Helicobacter*-like organisms. Treatment for *Helicobacter *spp. decreased the vomiting slightly. The dog was diagnosed with concurrent leishmaniosis; the conventional anti-*Leishmania* treatment decreased the vomiting moderately, the hypoalbuminaemia resolved and the dog gained weight back to a normal body condition. Granulomatous gastritis was not present in the gastric biopsies after these treatments. The dog increased vomiting when palliative treatment (maropitant citrate, ondansetron and esomeprazole) was discontinued, and thus, full-thickness biopsies of the stomach were taken and Ménétrier-like disease was diagnosed. The affected area was too large to be surgically removed; thus, palliative treatment was reinstated. The dog remained clinically well 39 months after the first clinical presentation.

**Conclusions:**

This is the first report of Ménétrier-like disease in a dog with a simultaneous manifestation of granulomatous gastritis, helicobacteriosis and leishmaniosis. The clinical signs decreased after treatment of helicobacteriosis and leishmaniosis, but vomiting remained probably as a sign of Ménétrier-like disease. Treatment options for dogs are surgical removal of the abnormal area or palliative treatment. In humans, promising results for a cure have been shown with cetuximab treatment, a human monoclonal antibody, but no canine antibody is commercially available yet. The dog here was doing well 39 months after first presentation, which is the longest reported survival time for Ménétrier-like disease with only palliative treatment in dogs. Full-thickness biopsies are preferred in macroscopic hypertrophic lesions of the stomach for better assessment of Ménétrier-like disease.

## Background

Ménétrier’s disease (MD) is a rare hypertrophic gastropathy in humans, characterized macroscopically by giant cerebriform gastric mucosal folds in the corpus and fundus of the stomach, sparing the antrum and pylorus [1, 2]. Histopathologically, hyperplasia of the mucous cells within the neck and base areas of the fundic glands and loss of parietal and chief cells are typical [1]. The criteria for diagnosis of MD in dogs have not yet been established in detail, which is why the term Ménétrier-like disease (MLD), instead of MD, is preferred in dogs. MLD has been reported earlier in only seven dogs representing five different breeds: a Boxer, an Old English Sheepdog, a West Highland White Terrier, three Cairn Terrier littermates and a Jack Russell Terrier [3–7]. In all reported canine cases, hyperplasia of the mucous cells in the corpus and fundus of the stomach was described, and in four cases also the loss of parietal and chief cells [6, 7]. The most common clinical signs and findings in affected dogs include vomiting, anorexia, weight loss and hypoalbuminaemia [3–7]. The aetiology underlying MLD in dogs is unknown, but in humans and mice, one factor in the pathogenesis of MD is suggested to be increased signalling of epidermal growth factor receptors (EGFR) [1]. In humans, MD usually shows chronic progressive features, but variants with acute onset and spontaneous remission have been reported, and have been associated with *Helicobacter pylori* infection in adults and cytomegalovirus (CMV) infection in children [2, 8]. This report describes for the first time the coexistence of MLD with granulomatous gastritis, a severe manifestation of *Helicobacter*-like organisms and leishmaniosis in a Pointer.

## Case presentation

A nine-year-old spayed female Pointer weighing 15 kg was presented with a nine-month history of intermittent vomiting and marked weight loss to the University of Helsinki Veterinary Teaching Hospital, Finland. The dog was born in Greece and had lived one year in Germany before moving to Finland six years before presentation. On physical examination, the dog´s body condition score was 3/9 and it had mild periodontitis.

Complete blood count results were within reference intervals. Serum biochemical abnormalities included mild hypoproteinaemia, mild hypoalbuminaemia and hypocobalaminaemia. C-reactive protein was mildly elevated. Antibody titres against *Leishmania infantum* antigens (immunofluorescence antibody test, Ludwig Maximilian University of Munich) were markedly elevated (Table 1). Serum gastrin concentration was 26 ng/l (reference interval 10–40 ng/l, Michigan State University). To rule out hypoadrenocorticism, basal serum cortisol concentration was measured and it was 34 nmol/l (reference interval 10–100 nmol/l, IDEXX Laboratories) [9]. Serum *Dirofilaria immitis* antigen and antibodies against *Borrelia burgdorferi*, *Anaplasma phagocytophilum* and *Ehrlichia canis* were negative (SNAP 4Dx, IDEXX Laboratories).

**Table 1:** Summary of changes in clinical biochemistry parameters and results of additional diagnostic tests

Serum parameter (reference interval)	FP	1 MAFP	2 MAFP	4 MAFP	7 MAFP	10 MAFP	17 MAFP	24 MAFP	32 MAFP	39 MAFP
Albumin (30–41 g/l)	**25.7**	NT	**26.1**	31.3	33.4	30.8	34.2	33.3	34.4	33.2
Total proteins (58–77 g/l)	**53.0**	NT	**57.0**	62.0	58.0	**57.0**	63.0	64.0	66.0	70.0
Cobalamin (200–850 pg/ml)	**163**	NT	737	1180	NT	**176**	766	839	904	725
C-reactive protein (< 10 mg/l)	**26.2**	**45.4**	< 10	NT	NT	NT	NT	NT	NT	NT
*Leishmania infantum* antibodies (< 1:32)	**1:512**	NT	NT	NT	NT	**1:256**	NT	NT	**1:64**	**1:64**

Values beyond the reference interval are bolded

*FP* First presentation, *MAFP* Months after the first presentation, *NT* Not tested

Faecal examination for endoparasites by flotation, antigen testing for *Giardia duodenalis* (FASTest Giardia) and bacterial culture for *Yersinia* spp., *Salmonella* spp., *Campylobacter* spp., *Clostridium difficile* and *Clostridium perfringens* were negative. Urinalysis including urine protein-creatinine ratio was unremarkable. Abdominal ultrasonography revealed a partial mild gastric wall thickening with slight loss of wall layering, mild thickening of the colonic wall with normal wall layering, enlarged and slightly heterogenic medial iliac lymph nodes, prominent mesenteric lymph nodes and hyperechoic sports in both renal cortices.

To exclude food-responsive enteropathy, an elimination diet was started with a hydrolysed diet in addition to palliative treatment comprising famotidine (Pepcid; Janssen-Cilag, 0.7 mg/kg orally q12h), ondansetron (Ondansetron Stada; Stada Arzneimittel, 0.3 mg/kg orally q8h) and maropitant citrate (Cerenia; Zoetis, 3.2 mg/kg orally q24h). Cobalamin supplementation was initiated (Betolvex; Ratiopharm, 0.06 mg/kg orally q24h). Treatment for leishmaniosis was not started until gastroduodenoscopy was performed and gastric biopsies examined.

**Fig. 1:**
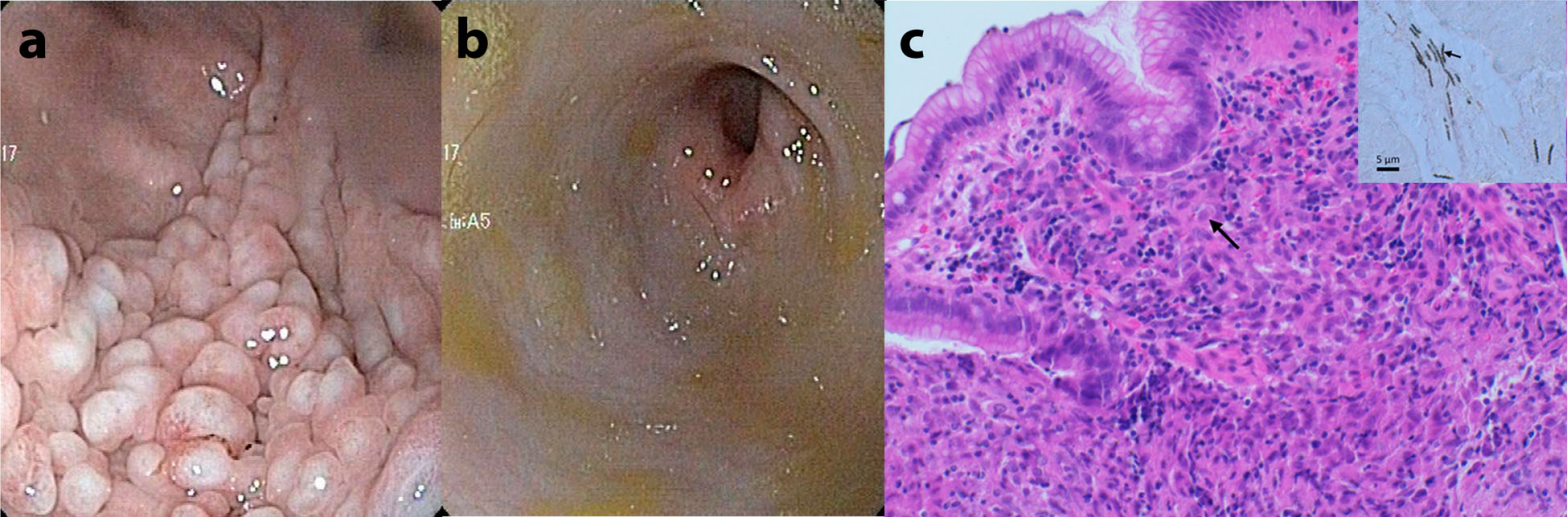
First gastroduodenoscopy. **a** Endoscopic view of gastric corpus and proximal antrum showing cerebriform thickened gastric mucosal folds. **b** Endoscopic view of distal antrum and pylorus was macroscopically unremarkable. **c** Histopathology of the gastric mucosal biopsy showing multifocal moderately increased number of macrophages (arrow), lymphocytes and plasma cells. Haematoxylin and eosin staining 20X. Insert: Numerous spiral-shaped *Helicobacter*-like organisms (arrow) are present in gastric mucosa. Warthin-Starry silver staining, bar 5 µm

Gastroduodenoscopy was performed one month after the first presentation since the dog did not improve after initiating treatment. The gastric mucosa of the corpus, fundus and proximal antrum was macroscopically irregular, showing cerebriform mucosal folds (Fig. 1a). The distal antrum (Fig. 1b) and duodenum were macroscopically unremarkable. Histopathological examination of the duodenum revealed a mild diffuse chronic lymphoplasmacytic enteritis with a mild multifocal neutrophilic component. Gastric mucosal biopsies showed a moderate multifocal chronic granulomatous gastritis with fewer lymphocytes and plasma cells (Fig. 1c) and occasional intralesional, round to ovoid, 3–6 µm in diameter single-cell organisms, which were detected both within the cytoplasm of macrophages and extracellularly. In addition, severe manifestation of *Helicobacter*-like organisms (HLOs) and multifocal mild cystic dilatation of glands at the midlevel of the lamina propria were observed. The organisms seen intralesionally stained positively in both periodic acid Schiff’s (PAS) and Grocott’s methenamine silver special stainings.

A PCR for fungi was performed on a fresh frozen gastric mucosal biopsy sample (HUSLAB), and DNA from *Malassezia globosa* was found [10]. *Leishmania* spp. was examined by immunohistochemistry from a formalin fixed gastric biopsy (Universitat Autònoma de Barcelona) and by real-time PCR from a fresh frozen gastric biopsy (IDEXX Laboratories); both were negative [11]. Fluorescence in situ hybridization (FISH) from a formalin fixed gastric biopsy detected eubacteria, but no significant amounts of *Escherichia coli*, *Salmonella* spp., *Helicobacter* spp., *Cryptococcus* spp., *Campylobacter* spp., *Leptospira* spp. or *Streptococcus* spp. (Langford Vets Diagnostic Laboratories).

Based on these findings, the dog was treated for helicobacteriosis with amoxicillin (Amovet vet; Orion, 25 mg/kg orally q12h), clarithromycin (Clarithromycin Ratiopharm; Teva, 8 mg/kg orally q12h) and metronidazole (Metronidazol Ratiopharm; Ratiopharm, 8 mg/kg orally q12h) for two weeks. The palliative treatment continued with ondansetron and maropitant citrate, in addition to esomeprazole (Nexium; AstraZeneca, 1 mg/kg orally q24h). Oral supplementation of cobalamin was also continued.

**Fig. 2:**
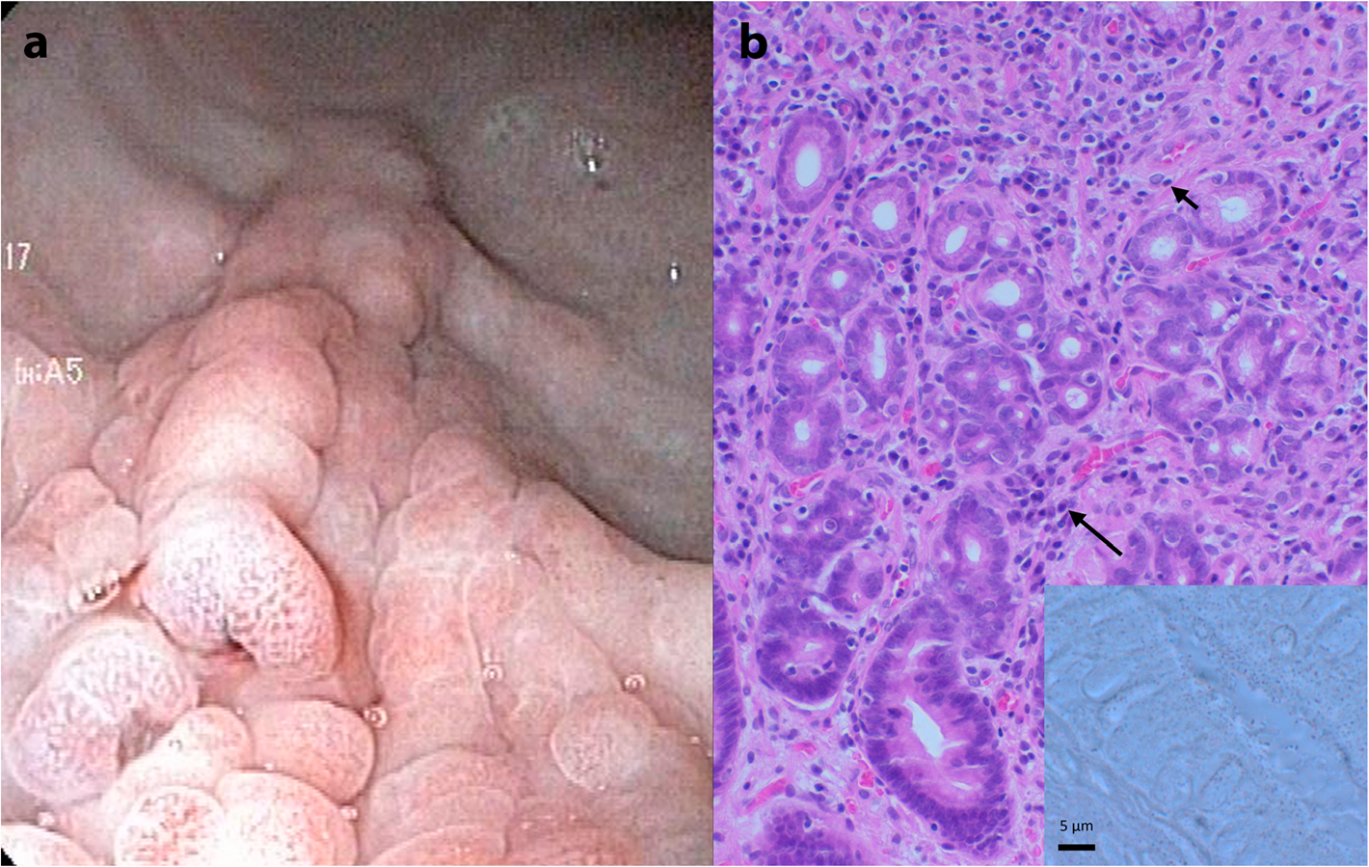
Second gastroduodenoscopy. **a** Endoscopic view of the gastric corpus and proximal antrum showing the same giant cerebriform gastric mucosal folds as in the first endoscopy. **b** Histopathology of the gastric mucosal biopsy after treatment for *Helicobacter*-like organisms revealing a severe chronic diffuse lymphoplasmacytic gastritis (lymphocytes short arrow, plasma cells long arrow) with a decreased number of macrophages relative to the first gastric biopsies. Haematoxylin and eosin staining 20X. Insert: No *Helicobacter*-like organisms were detected. Warthin-Starry silver staining, bar 5 µm

One month later, the dog still vomited every other day. A second gastroduodenoscopy was performed. The gastric mucosal changes had the same magnitude and cerebriform appearance as in the first gastroduodenoscopy (Fig. 2a). Histopathological examination revealed a severe diffuse chronic lymphoplasmacytic gastritis with fewer macrophages. Some single-cell organisms were still present, but HLOs were no longer detected (Fig. 2b). The palliative treatment was continued. Based on the high antibodies against *L. infantum* antigens and persistent clinical signs, treatment for leishmaniosis was started according to LeishVet guidelines [12] with miltefosine (Milteforan; Virbac, 2 mg/kg orally q24h for 4 weeks) and allopurinol (Zyloric; Aspen, 10 mg/kg orally q12h).

**Fig. 3:**
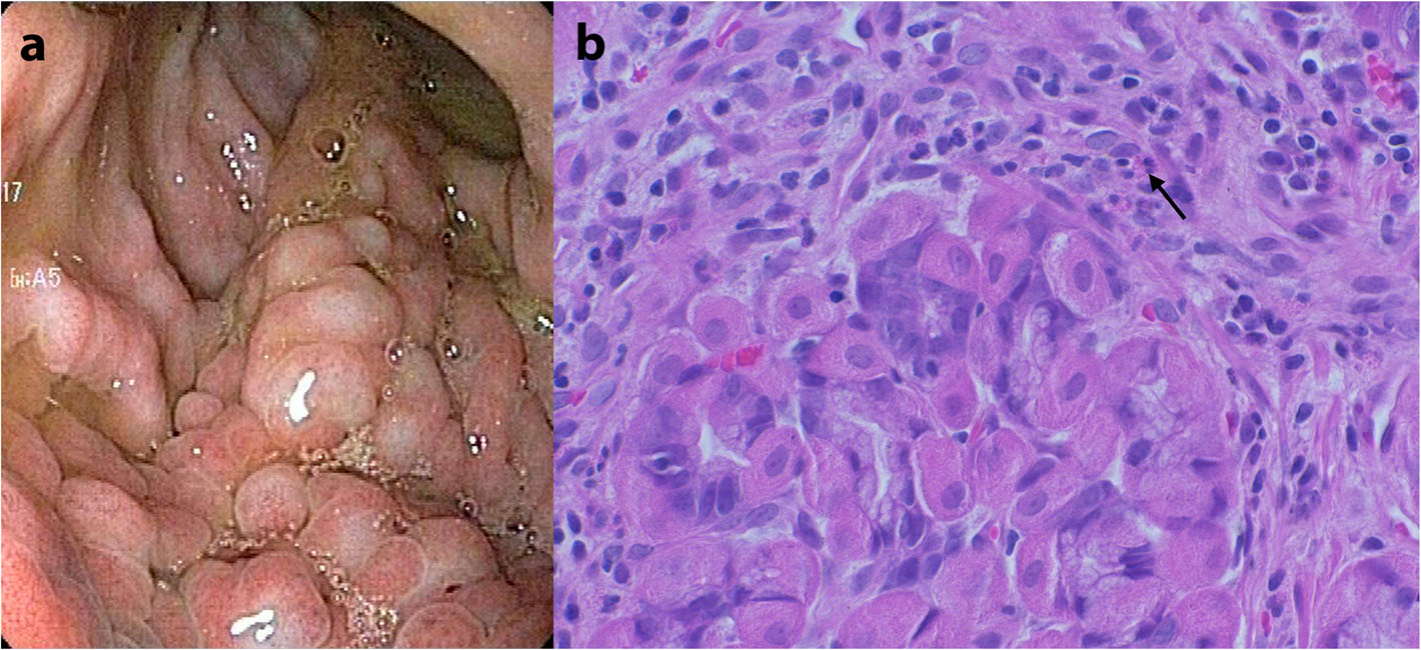
Third gastroduodenoscopy. **a** Endoscopic view of the gastric corpus and proximal antrum showing the same giant cerebriform gastric mucosal folds as in the previous endoscopies. **b** Histopathology of the gastric mucosal biopsy revealed fundic glands surrounded by eosinophilic granulocytes (arrow), lymphocytes and plasma cells. Haematoxylin and eosin staining 40X.

One month later, the vomiting had stopped and the dog had gained weight. Serum total protein and albumin concentrations increased to the reference intervals, after which the oral cobalamin supplementation was discontinued. A third gastroduodenoscopy was performed. The macroscopically changed mucosal area was the same size and had the same appearance as during the first two endoscopies, but the mucosa was somewhat firmer (Fig. 3a). Histopathological examination now revealed a moderate diffuse chronic eosinophilic gastritis (Fig. 3b). Mild granulomatous inflammation was noted only focally in the corpus area. Single-cell organisms seen previously were no longer detected, but a small number of HLOs was seen. The palliative treatment and allopurinol were continued.

**Fig. 4:**
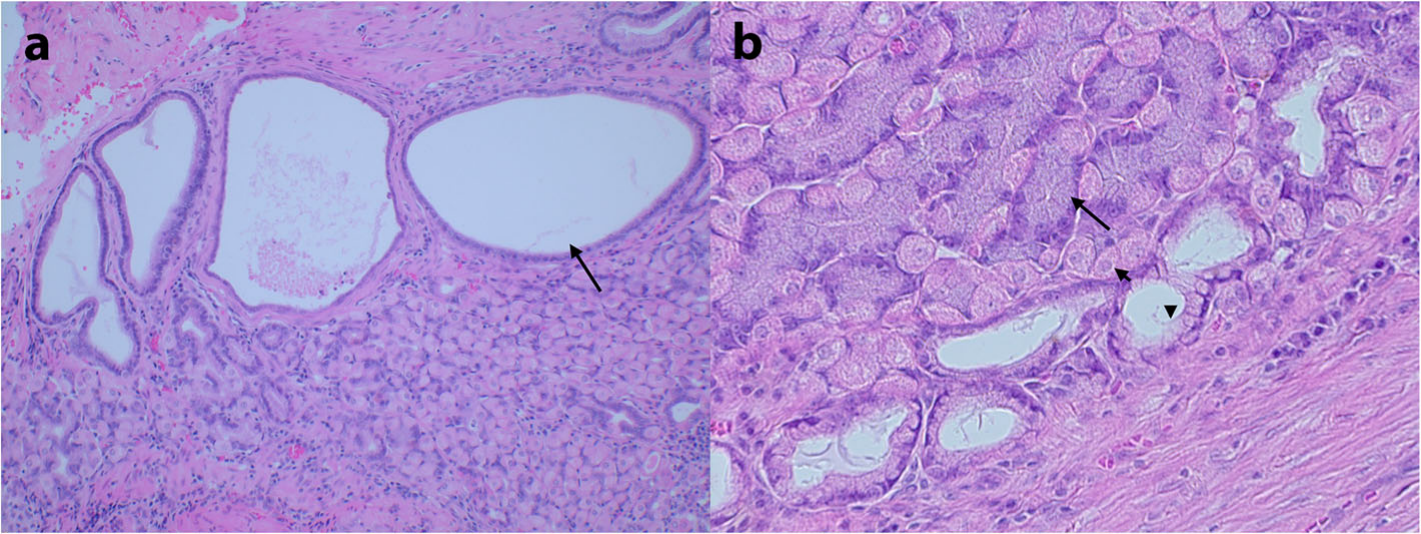
Histopathology of the full-thickness biopsies of the fundus showing histopathological features of MD. **a** Cystic enlargement of mucinous glandular structures (arrow). Haematoxylin and eosin staining 10X. **b** Hyperplasia of the mucous cells (arrow head) and loss of parietal cells (short arrow) and chief cells (long arrow). Haematoxylin and eosin staining 40X.

Seven months after the first presentation, the dog showed signs of heat for the first time after being spayed seven years earlier in Greece and was diagnosed ultrasonographically with an ovarian remnant on the left side. A laparotomy was performed and the ovarian remnant was removed and two full-thickness biopsies were taken from the gastric fundus and antrum. The aerobic and anaerobic bacterial culture of the gastric biopsies was negative. Histological examination of the fundic biopsy showed hyperplasia of the mucous cells within the neck and base areas of the fundic glands, multifocal cystic enlargement of mucinous glandular structures at the border between the lamina propria and lamina muscularis mucosae and loss of parietal and chief cells (Fig. 4). Occasional glandular structures were growing between the muscular bundles on the lamina muscularis mucosae. The fundic glandular changes were consistent with the histopathological findings described in MD. A mild multifocal granulomatous inflammation in the submucosa remained. There were no specific histopathological changes in the antral biopsy.

The dog had gained weight, achieving a normal body condition score (5/9), and with palliative treatment and allopurinol it had only vomited approximately twice a month. The palliative medications continued, but at a lower dose, with esomeprazole 0.7 mg/kg orally q24h, maropitant citrate 0.8 mg/kg orally q24h and ondansetron 0.3 mg/kg orally q24h. If any of the palliative medications were stopped or titrated down, the dog started vomiting again daily.

Ten months after the first examination, the dog developed hypocobalaminaemia again (Table 1), so cobalamin was started orally at 0.03 mg/kg q24h. Oral cobalamin supplementation was successful again (Table 1). Serum albumin concentration remained within the reference interval. Thirty-nine months after the first presentation, the dog was still on allopurinol, ondansetron and maropitant citrate and was vomiting approximately twice a month.

## Discussion and conclusions

This case report describes the coexistence of two rare gastropathies in the dog, namely MLD and granulomatous gastritis, with simultaneous helicobacteriosis and leishmaniosis. The pathogenesis of MLD in dogs is unknown, but in MD in humans and mice, one factor is suggested to be increased signalling of EGFR [1]. Especially in children, MD is strongly associated with CMV infection, and CMV has been shown to activate EGFR signalling [2]. In CMV-associated cases, there is a spontaneous remission of MD [2]. Especially in adults, some MD cases have been associated with *H. pylori* infection and the eradication of *H. pylori* has resolved the endoscopic and histopathological findings [8]. In this dog, a severe manifestation of HLOs was present in the first mucosal biopsies of the stomach, which has not previously been reported in MLD cases in dogs [3–7].

The dog showed chronic upper gastrointestinal signs, such as weight loss, anorexia and vomiting in connection with hypoalbuminaemia, which are typical clinical signs for MLD in dogs and MD in humans [1, 3–5, 7]. In this case, vomiting slightly decreased when palliative treatment was initiated and later slightly more with treatment for *Helicobacter* spp., but it did not cease. The *Helicobacter* spp. treatment also did not result in resolution of MLD. This could be explained with the chronic features of the dogs’ disease. *H. pylori* –associated MD in humans going into remission, typically has an acute onset of symptoms [2]. After treatment for leishmaniosis, the vomiting ceased, the dog gained weight and the hypoalbuminaemia resolved. It seems likely that part of the clinical signs were caused by leishmaniosis. Six months after the miltefosine treatment, the antibody titres against *L. infantum* had halved, indicating that the leishmaniosis was in remission. The dog always started vomiting if any of the palliative treatment medications: maropitant citrate, ondansetron or esomeprazole, were stopped or titrated down. At this point, the vomiting was probably a sign of MLD.

In humans, treatments other than *H. pylori* eradication for MD have also been tried, such as antibiotics, steroids, acid suppressants, anticholinergic agents and octreotide therapy, but the findings have been inconsistent [2, 13]. In humans, promising results have been shown with cetuximab, a human monoclonal antibody that blocks EGFR signalling [1]. A “caninized” anti-EGFR antibody has been investigated only *in vitro* in canine cancer, however, no canine anti-EGFR antibody is commercially available. Human anti-EGFR antibodies could cause adverse reactions in dogs such as anaphylaxis [14]. Partial or total gastrectomy has been done in cases with severe symptoms, and it has been reported to be a successful treatment also in two canine cases [2, 5, 7, 13]. In this case, the affected area was too large to be removed surgically without affecting the function of the stomach; palliative treatment was given since the dog responded to it. Thirty-nine months after first presentation, the dog was clinically well, vomiting approximately twice a month, despite the low daily doses of the palliative treatments. Previously, the longest reported survival time in a dog not treated surgically was 27 months [6]. That dog had a subclinical disease for two years, but was given a gastric protectant, a proton pump inhibitor and a non-steroidal anti-inflammatory drug for an unknown period [6].

The dog here was hypocobalaminaemic and was supplemented orally with cobalamin [15]. The supplementation led to hypercobalaminaemia and was discontinued, after which the dog developed hypocobalaminaemia again. In dogs, aetiologies for hypocobalaminaemia are exocrine pancreatic insufficiency, chronic inflammatory enteropathies and alimentary lymphoma among others [16]. This dog showed no evidence of these diseases. In the stomach, free cobalamin is bound to haptocorrin, which in dogs is mostly produced by parietal cells in the gastric mucosa [17]. Haptocorrin is digested by pancreatic proteases in the duodenum and then free cobalamin is bound to intrinsic factor [16]. Intrinsic factor is mainly produced in dogs by the exocrine pancreas, but also in the stomach in parietal cells [16, 17]. Parietal cells, which produce both haptocorrin and intrinsic factor, are typically lost in MD [1, 16, 17], which could be one possible factor contributing to the hypocobalaminaemia in this dog.

The serum gastrin concentration was measured to rule out gastrinoma as a differential diagnose for the hypertrophic changes in the stomach. The typical changes of MLD were not evident in the endoscopic gastric mucosal biopsies, but the diagnosis was achieved from the full-thickness biopsies. In humans, the diagnosis for MD is obtained from endoscopic biopsies, but large snare biopsies are recommended in macroscopically hypertrophic lesions, instead of standard forceps biopsies [2]. In the previous canine case reports, the diagnosis was made from full-thickness biopsies in all cases [3–7]. In one case, the first biopsies were taken by gastroscopy and they had contained only the superficial part of the mucosa from which the diagnosis of MLD could not be made [3]. In the case presented here, it is uncertain why the characteristic changes of MLD were not seen in the first three endoscopic mucosal biopsies because all samples had contained the lamina propria of the gastric mucosa and the macroscopic appearance of the gastric lesion at the endoscopies and the laparotomy had remained the same. Based on this, full-thickness biopsies should probably be the preferred method in dogs with suspected MLD. Full-thickness biopsies are also more accurate for the evaluation of possible neoplastic changes. People with MD have a higher risk of developing gastric neoplasia [1]. Four cases of MLD in dogs have been reported to progress to gastric carcinoma [5, 6]. In three littermates, gastric adenocarcinoma was diagnosed [6]. In two of the dogs, the diagnosis was set at the same time as the diagnosis of MLD [6]. In the third dog, the diagnosis was made 27 months after diagnosis of MLD [6]. In one dog, a poorly differentiated superficial gastric carcinoma was diagnosed five years after a partial gastrectomy was done and MLD was diagnosed [5].

Granulomatous gastritis in dogs is far less common than lymphoplasmacytic or eosinophilic gastritis and has been described in rare cases of infections caused by fungi, parasites or bacteria [18]. The cause for the granulomatous gastritis in this dog was extensively examined, yet remained unknown. Granulomatous inflammation in the gastrointestinal tract of dogs in endemic areas of leishmaniosis has been reported in *L. infantum* infection [19]. However, *L. infantum* was not detected in the gastric biopsies by PCR or immunohistochemistry. The aerobic and anaerobic bacterial culture of the biopsy was negative. The PCR examination for fungi revealed a small amount of DNA from *M. globosa*. The PAS-positive intralesional structures in the stomach were morphologically compatible with single-cell yeast. *Malassezia* spp. was not thought to be the cause for the granulomatous gastritis. The PAS-positive structures also disappeared from the biopsies without a treatment for *Malassezia* spp. FISH detected eubacteria within the gastric tissue samples, but not significant numbers of *E. coli*, *Salmonella* spp., *Helicobacter* spp., *Cryptococcus* spp., *Campylobacter* spp., *Leptospira* spp. or *Streptococcus* spp. It is unclear why the severe manifestation of HLO was not detected by FISH since in previous studies *Helicobacter* spp. have been detected by FISH with high sensitivity [20, 21]. In one study, *Helicobacter* spp. was detected by FISH in all gastric biopsies, but in a few biopsies from the small and large intestine they were not detected by FISH, even though *Helicobacter* spp. PCR had been positive [20]. This was thought to be associated with either a difficulty of the probes in penetrating the crypts or too few bacteria in the sample [20]. This could also be the case in our biopsies.

The role of *Helicobacter* spp. in the granulomatous gastritis of this dog is unknown. Typically, *Helicobacter* spp. infection in dogs manifests as mild lymphoplasmacytic gastritis, but can also be asymptomatic [22, 23]. In some human cases, it has been suggested that there might be an association between *H. pylori* infection and granulomatous gastritis [24, 25]. In this case, the severity of the granulomatous gastritis markedly decreased after the treatment for *Helicobacter* spp, which supports a possible connection between the two conditions. However, we cannot exclude a response to the simultaneous treatment of leishmaniosis. Two months after the treatment, *Helicobacter* spp. were detected again in the gastric biopsies. Recurrence of gastric *Helicobacter* spp. after eradication is a common finding in dogs [26].

In conclusion, the dog was diagnosed with MLD, granulomatous gastritis, severe helicobacteriosis and leishmaniosis. The clinical signs decreased after treatment of helicobacteriosis and leishmaniosis, but mild chronic vomiting remained probably as a sign of MLD. The dog was doing well 39 months after the first presentation, which is the longest reported survival time for canine MLD with palliative treatment. Full-thickness biopsies are valuable in macroscopic hypertrophic lesions of the stomach for better assessment of the disease.

## Data Availability

All data are presented in the main paper and the accompanying figures.

## Abbreviations

CMV: Cytomegalovirus
EGFR: Epidermal growth factor receptor
FISH: Fluorescence in situ hybridization
HLO: *Helicobacter*-like organism
MD: Ménétrier’s disease
MLD: Ménétrier-like disease
PAS: Periodic acid Schiff’s staining

## References

[1] Fiske WH, Tanksley J, Nam KT, Goldenring JR, Slebos RJ, Liebler DC (2009). Efficacy of cetuximab in the treatment of Ménétrier’s disease. Sci Transl Med.

[2] Huh WJ, Coffey RJ, Washington MK (2016). Ménétrier’s disease: its mimickers and pathogenesis. J Pathol Transl Med..

[3] Van der Gaag I, Happé RP, Wolvekamp WT (1976). A boxer dog with chronic hypertrophic gastritis resembling Menetrier’s disease in man. Vet Pathol.

[4] Rallis TS, Patsikas MN, Mylonakis ME, Day MJ, Petanides TA, Papazoglou LG (2007). Giant hypertrophic gastritis (Ménétrier’s-like disease) in an Old English sheepdog. J Am Anim Hosp Assoc.

[5] Lecoindre P, Bystricka M, Chevallier M, Peyron C (2012). Gastric carcinoma associated with Menetrier’s-like disease in a West Highland white terrier. J Small Anim Pract.

[6] Munday JS, Aberdein D, Cullen GD, French AF (2012). Ménétrier disease and gastric adenocarcinoma in 3 Cairn terrier littermates. Vet Pathol.

[7] Vaughn DP, Syrcle J, Cooley J (2014). Canine giant hypertrophic gastritis treated successfully with partial gastrectomy. J Am Anim Hosp Assoc.

[8] Badov D, Lambert JR, Finlay M, Balazs ND (1998). *Helicobacter pylori* as a pathogenic factor in Ménétrier’s disease. Am J Gastroenterol.

[9] Gold AJ, Langlois DK, Refsal KR (2016). Evaluation of basal serum or plasma cortisol concentrations for the diagnosis of hypoadrenocorticism in dogs. J Vet Intern Med.

[10] Ala-Houhala M, Koukila-Kähkölä P, Antikainen J, Valve J, Kirveskari J, Anttila VJ (2018). Clinical use of fungal PCR from deep tissue samples in the diagnosis of invasive fungal diseases: a retrospective observational study. Clin Microbiol Infect.

[11] Casanova MI, Martín S, Marco A, Solano-Gallego L (2019). Detection of *Leishmania* spp. infection by immunohistochemistry in archived biopsy samples from dogs with colitis in an area endemic for leishmaniosis. J Comp Pathol.

[12] Solano-Gallego L, Mirá G, Koutinas A, Cardoso L, Pennisi MG, Ferrer L (2011). LeishVet guidelines for the practical management of canine leishmaniosis. Parasit Vect.

[13] Rich A, Toro TZ, Tanksley J, Fiske WH, Lind CD, Ayers GD (2010). Distinguishing Ménétrier’s disease from its mimics. Gut.

[14] Singer J, Fazekas J, Wang W, Weichselbaumer M, Matz M, Mader A (2014). Generation of a canine anti-EGFR (ErbB-1) antibody for passive immunotherapy in dog cancer patients. Mol Cancer Ther.

[15] Toresson L, Steiner JM, Suchodolski JS, Spillmann T (2016). Oral cobalamin supplementation in dogs with chronic enteropathies and hypocobalaminemia. J Vet Intern Med.

[16] Kather S, Grützner N, Kook PH, Dengler F, Heilmann RM (2020). Review of cobalamin status and disorders of cobalamin metabolism in dogs. J Vet Intern Med.

[17] Seetharam B, Alpers DH, Dakshinamurti K (1994). Cobalamin binding proteins and their receptors. Vitamin receptors: vitamins as ligands in cell communication.

[18] Amorim I, Taulescu MA, Day MJ, Catoi C, Reis CA, Carneiro F, Gärtner F (2016). Canine gastric pathology: a review. J Comp Pathol.

[19] Ruiz G, Laloy E, Benchekroun G (2015). Chronic gastritis and enterocolitis associated with *Leishmania* infection in an 18-month-old, intact female dog. Vet Q.

[20] Recordati C, Gualdi V, Craven M, Sala L, Luini M, Lanzoni A, Rishniw M, Simpson KW, Scanziani E (2009). Spatial distribution of *Helicobacter* spp. in the gastrointestinal tract of dogs. Helicobacter.

[21] Sharman M, Bacci B, Simpson K, Mansfield C (2016). Comparison of *in vivo* confocal endomicroscopy with other diagnostic modalities to detect intracellular helicobacters. Vet J.

[22] Neiger R, Simpson KW (2000). *Helicobacter* infection in dogs and cats: facts and fiction. J Vet Intern Med.

[23] Wiinberg B, Spohr A, Dietz HH, Egelund T, Greiter-Wilke A, McDonough SP (2005). Quantitative analysis of inflammatory and immune responses in dogs with gastritis and their relationship to *Helicobacter* spp. infection. J Vet Intern Med.

[24] Kim YS, Lee HK, Kim JO (2009). A case of *H. pylori*-associated granulomatous gastritis with hypertrophic gastropathy. Gut Liver.

[25] Koyama S, Nagashima F (2003). Idiopathic granulomatous gastritis with multiple aphthoid ulcers. Intern Med.

[26] Happonen I, Linden J, Westermarck E (2000). Effect of triple therapy on eradication of canine gastric helicobacters and gastric disease. J Small Anim Pract.

